# Supplementary material for: Spherical Polyelectrolyte Brushes as Templates to Prepare Hollow Silica Spheres Encapsulating Metal Nanoparticles
Source: Nanomaterials (Basel). 2020 Apr 21;10(4):799. doi: 10.3390/nano10040799 (PMC7221898; doi:10.3390/nano10040799)
Supplement: Supplementary file 1 [file nanomaterials-10-00799-s001.pdf]

Supplementary Information

Spherical Polyelectrolyte Brushes as Templates to Prepare Hollow Silica Spheres Encapsulating Metal Nanoparticles

Qingsong Yang [1], Li Li [1,*], Fang Zhao [1], Yunwei Wang [1], Zhishuang Ye [1], Chen Hua [1], Zhiyong Liu [2], Klemen Bohinc [3] and Xuhong Guo [1,2,*]

[1] State Key Laboratory of Chemical Engineering, School of Chemical Engineering, East China University of Science and Technology, 200237 Shanghai, P.R. China; yangqingsong@mail.ecust.edu.cn (Q.Y.); fzhao1@ecust.edu.cn (F.Z.); yunweiwang@mail.ecust.edu.cn (Y.W.); zhishuangye@mail.ecust.edu.cn (Z.Y.); huachecust@gmail.com (C.H.)

[2] Engineering Research Center of Materials Chemical Engineering of Xinjiang Bingtuan, Shihezi University, 832000 Xinjiang, P.R. China; lzy_tea@shzu.edu.cn (Z.L.)

[3] Faculty of Health Sciences, University of Ljubljana, 1000 Ljubljana, Slovenia; bohinck@zf.uni-lj.si (K.B.)

* Correspondence: lili76131@ecust.edu.cn (L.L.); guoxuhong@ecust.edu.cn (X.G.); Tel.: +86-21-64253789 (L.L.); +86-21-6425-3491 (X.G.)

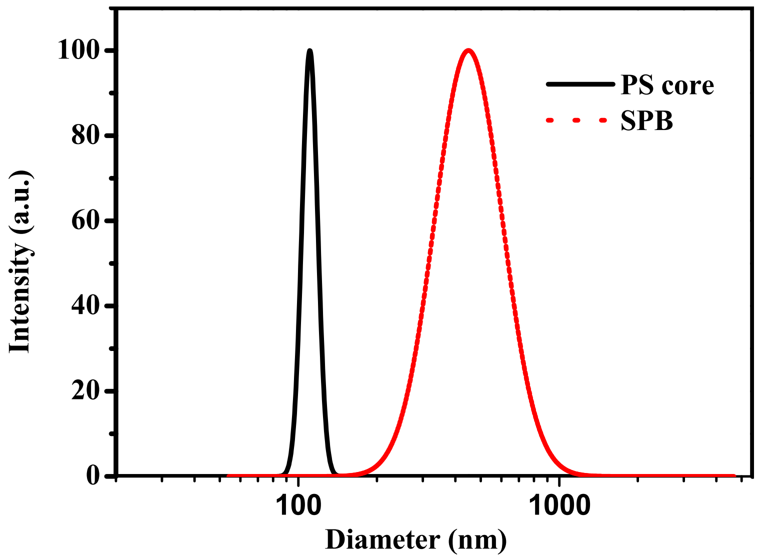

Figure S1. Hydrodynamic size and size distribution of PS core and SPB determined by DLS at pH = 7 and [NaCl] = 10 mM.

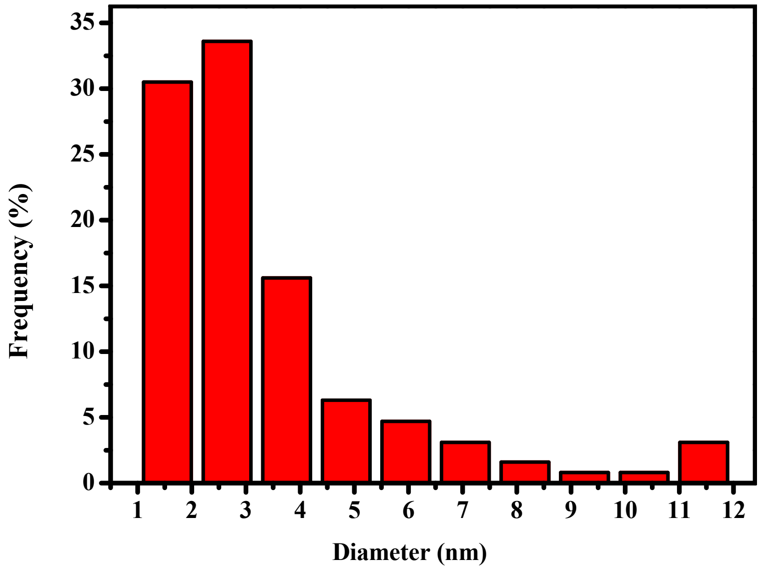

Figure S2. Size histograms of the diameters of encapsulated silver nanoparticles of $Ag@SiO_2$ spheres.
